# Prophylactic pyloric drainage for prevention of delayed gastric conduit emptying following Ivor Lewis esophagectomy: a systematic review with exploratory synthesis

**DOI:** 10.1007/s00464-026-13189-3

**Published:** 2026-07-31

**Authors:** Pascal Burri, Dimitrios Chatziisaak, Marcel A. Schneider, Christian A. Gutschow, Kristjan Ukegjini, René Warschkow, Thomas Steffen

**Affiliations:** 1 Department of Visceral and Transplant Surgery, University Hospital Zurich, CH-8006 Zurich, Switzerland; 2 Department of Surgery, HOCH St.Gallen, CH-9007 St. Gallen, Switzerland; 3 Département de Chirurgie, Centre Hospitalier Universitaire Vaudois, CH-1005 Lausanne, Switzerland; 4 Department of Transplantation Surgery, Guy’s and St Thomas’ NHS Foundation Trust, London, UK; 5 Nova Chirurgie, CH-9016 St. Gallen, Switzerland

**Keywords:** DGCE, Ivor Lewis esophagectomy, Pyloric drainage procedures, Pyloroplasty, Botulinum toxin, Postoperative complications

## Abstract

**Background:**

DGCE complicates 10–50% of ILE and is associated with prolonged hospital stay, aspiration pneumonia, and impaired quality of life. Several intraoperative drainage techniques have been proposed, but the optimal prophylactic approach remains unclear.

**Methods:**

We searched MEDLINE, Embase, Scopus, and Cochrane (PRISMA 2020; PROSPERO CRD420251025012; SWiM reporting guideline) for studies comparing intraoperative prophylactic pyloric drainage (pyloroplasty, botulinum toxin injection, pyloromyotomy, or mechanical balloon dilation) with no intervention in ILE. Risk of bias was assessed using RoB 2 (randomized trials) and ROBINS-I (non-randomized studies), and certainty of evidence was assessed using the GRADE framework. Pooled estimates were framed as exploratory.

**Results:**

Four studies (*n* = 739; one RCT, three observational cohorts) were eligible. Pooled DGCE in the no-intervention arms was 32.1% (95% CI 4.4–82.9%; *I*^2^ = 93.7%). Exploratory random-effects risk ratios versus no intervention were 0.28 (95% CI 0.03–2.24) for pyloroplasty (*k* = 3) and 0.71 (95% CI 0.48–1.05) for botulinum toxin (*k* = 3); excluding Cerfolio 2009 (POD-4 radiologic outcome incompatible with the other studies), the estimates were 0.07 (95% CI 0.01–0.59) and 0.99 (95% CI 0.54–1.81), respectively. Pyloromyotomy is reported in a single study and described narratively. Certainty of evidence (GRADE): very low.

**Conclusion:**

The current evidence is too sparse and heterogeneous to support routine intraoperative pyloric drainage in ILE. Use of standardized DGCE definitions and adequately powered ILE-restricted randomized trials is required.

**Graphical abstract:**

**Figure:**
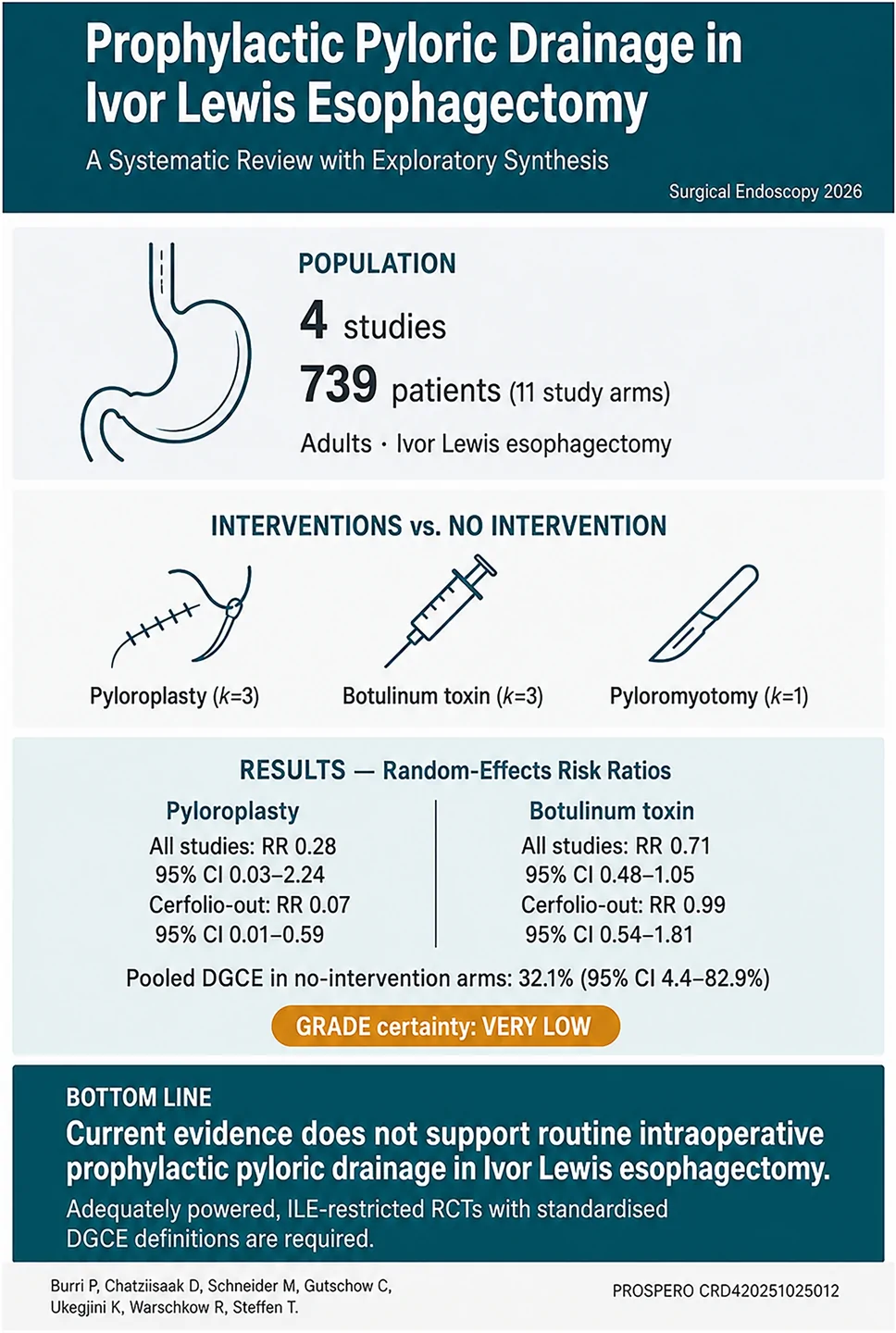

**Supplementary Information:**

The online version contains supplementary material available at https://doi.org/10.1007/s00464-026-13189-3.

Surgical resection with gastric conduit reconstruction remains the cornerstone of curative treatment in patients with esophageal cancer [1]. DGCE is a frequent postoperative complication, affecting 10–50% of patients [2–4]. Clinically, DGCE manifests as nausea, vomiting, and early satiety, delays resumption of oral intake, and increases the risk of serious complications such as aspiration pneumonia [5]. Impaired digestive function significantly impacts long-term quality of life [6], and preventing such complications is vital to facilitate timely adjuvant therapy, which is associated with improved oncologic outcomes [7].

The etiology of DGCE is multifactorial. While the prevailing hypothesis attributes it to neural dysfunction and pyloric spasm resulting from intraoperative vagotomy [8–10], recent evidence suggests a more complex, clinically relevant classification of DGCE. This includes distinguishing between functional pyloric obstruction and mechanical factors related to the conduit itself, such as redundant length, axis rotation, compression at the diaphragmatic hiatus, and the unfavorable pressure gradient between the thorax and abdomen [11].

To address the functional component of DGCE, various intraoperative pyloric drainage techniques have been employed. These include chemical interventions such as botulinum toxin (Botox) injection [12] and mechanical methods ranging from the Heineke-Mikulicz pyloroplasty and pyloromyotomy [13] to manual (pyloroclasy) or pneumatic dilation [14]. The efficacy of these procedures remains controversial [5]. Pyloric interventions may prolong operative time and potentially increase the risk of complications, including impaired conduit perfusion, dumping syndrome, bile reflux, and postoperative leakage [15].

Despite the historical use of various mechanical widening techniques, there is no current consensus on the optimal approach. This systematic review with exploratory synthesis aims to evaluate the efficacy of intraoperative pyloric drainage procedures in reducing the incidence of DGCE following ILE and to ascertain which, if any, technique provides the most robust clinical benefit. This review focuses exclusively on ILE with intrathoracic anastomosis, as dysphagia related to a cervical anastomosis may mimic impaired gastric emptying and confound outcome assessment [16].

## Materials and methods

### Literature search

**Fig. 1:**
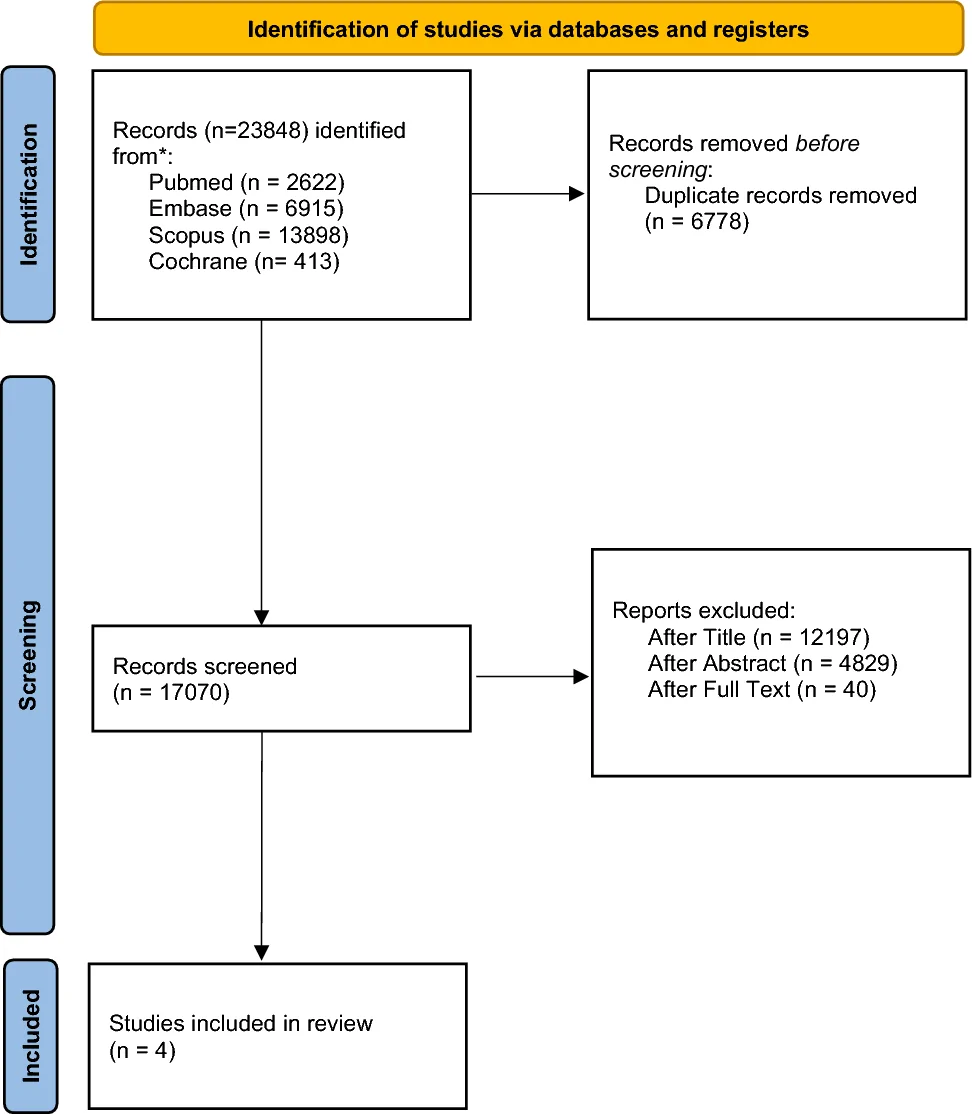
PRISMA 2020 flow diagram for new systematic reviews which included searches of databases and registers only. *Consider, if feasible to do so, reporting the number of records identified from each database or register searched (rather than the total number across all databases/registers). **If automation tools were used, indicate how many records were excluded by a human and how many were excluded by automation tools. Source: Page MJ, et al. BMJ 2021;372:n71. https://doi.org/10.1136/bmj.n71. This work is licensed under CC BY 4.0. To view a copy of this license, visit

This systematic review adhered to the PRISMA guidelines (Fig. 1) [17–19]. The protocol for this review was prospectively registered in the PROSPERO under the registration number CRD420251025012. The comprehensive literature search was conducted in the electronic databases MEDLINE (via PubMed), Embase, Scopus, and Cochrane, covering the period from January 1, 1945, to October 2, 2025. The search strategy was developed using Boolean operators and MeSH and is described in detail in Supplementary Appendices 1–4.

### Inclusion and exclusion criteria

**Fig. 2:**
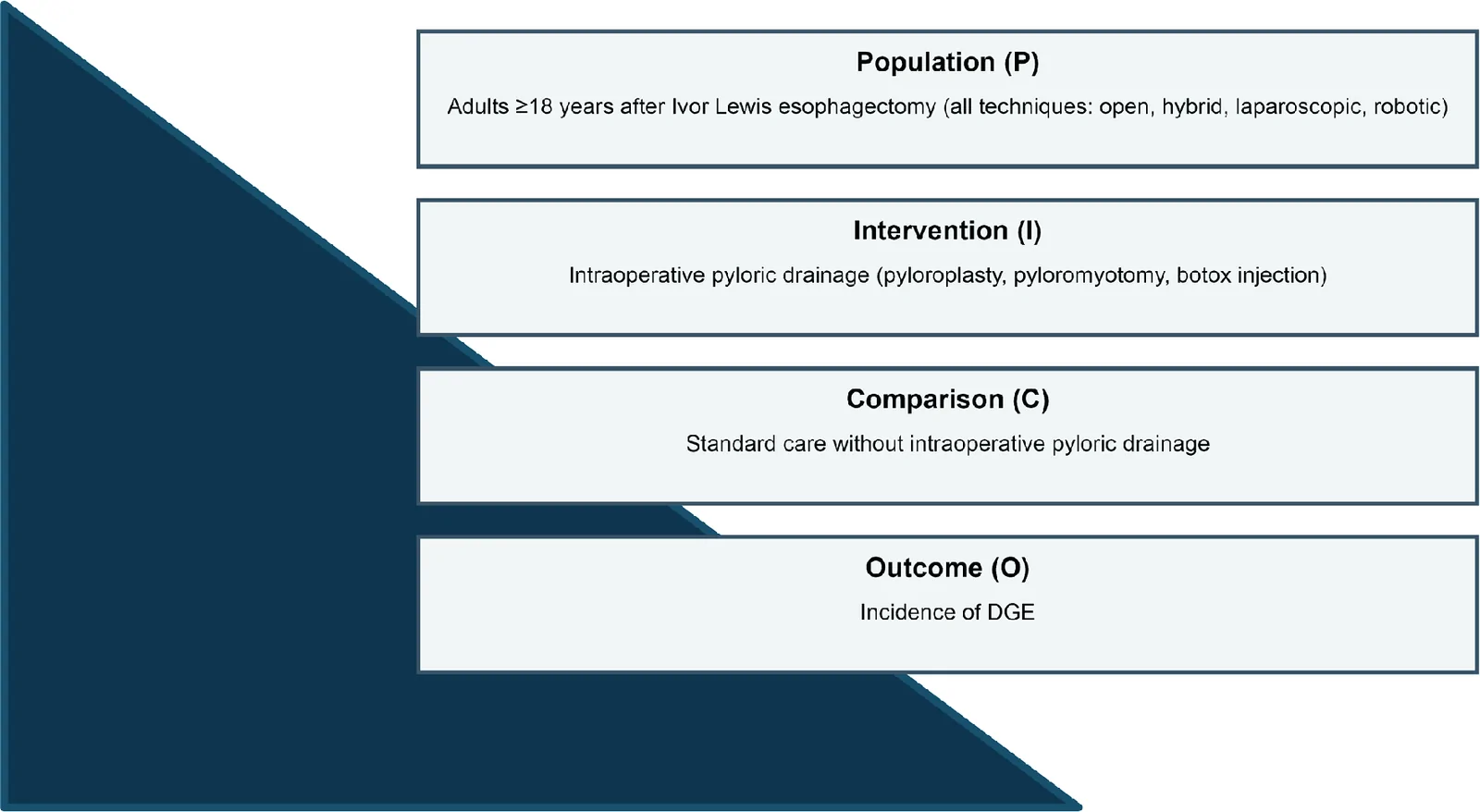
PICO framework defining the review’s eligibility criteria. The population (P), intervention (I), comparison (C), and outcome (O) domains used to structure the systematic search and study selection for intraoperative prophylactic pyloric drainage versus no drainage in ILE

Eligibility criteria were defined according to the PICO framework, encompassing the population, intervention, comparison, and outcome, as illustrated in Fig. 2 [20]. Study selection was performed in two stages: initial screening of titles and abstracts, followed by full-text review of eligible studies. Data extraction was undertaken independently by three reviewers (P.B., D.C., K.U.), with discrepancies resolved by consensus involving a fourth reviewer (T.S.).

The primary outcome was the incidence of DGCE. Owing to the absence of a standardized definition, all author-defined DGCE criteria were accepted, including clinical, radiological, and nasogastric tube-based definitions (Table 1). Where multiple definitions or time points were reported, the primary or most clinically relevant outcome was extracted, with preference given to early postoperative data. Notably, most included studies predate the standardized diagnostic criteria proposed by Konradsson (2020), which likely contributes to the substantial heterogeneity observed in this analysis [21].

**Table 1:** Study characteristics

Study	Country	Design	Center	Total N	Study arms	Age (years)	Male sex (%)	Histology	Neoadjuvant therapy (%)	Surgical approach	DGCE definition	DGCE assessment	Timing of DGCE assessment	Follow-up duration
Fok et al. (1991)	Hong Kong	Prospective RCT	Single-center	200	Pyloroplasty (*n* = 100); No Intervention (*n* = 100)	62 / 60	181 (90.5%)	NR	NR	Open	Clinical gastric outlet obstruction (GOO): symptoms with radiologic evidence of gastric dilatation and persistent regurgitation requiring TPN	Clinical + Radiologic	Clinical, no fixed time-point (any time during follow-up)	10 months (median survival)
Cerfolio et al. (2009)	USA	Retrospective cohort	Single-center	221	Pyloroplasty (*n* = 28); Pyloromyotomy (*n* = 71); No Intervention (*n* = 54); Botulinum toxin (*n* = 68)	64 / 56 / 58 / 64	139 (62.9%)	NR	NR	Open	Delayed gastric emptying on POD 4 swallow study	Radiologic	Postoperative day 4 (early inpatient)	40 months (median)
Marchese et al. (2018)	UK	Prospective cohort	Multi-center	90	No Intervention (*n* = 30); Botulinum toxin (*n* = 30); Pyloroplasty (*n* = 30)	68 / 65 / 68	72 (80.0%)	NR	90 (100%)	NR (open or hybrid)	DGCE symptoms at first outpatient follow-up: persistent postprandial vomiting within 2 h or NGT output > 500 mL/24 h	Clinical	First outpatient follow-up after discharge (post-discharge)	10 months (approx)
Tham et al. (2019)	UK	Retrospective cohort	Single-center	228	Botulinum toxin (*n* = 65); No Intervention (*n* = 163)	69 / 69	176 (77.2%)	AC 135; SCC 23	146 (64.0%)	Hybrid (open + minimally invasive)	Algorithm-based DGCE: NG-tube input/output combined with chest X-ray appearance per the local enhanced-recovery protocol	Clinical + Radiologic (algorithmic)	During index admission (early inpatient, protocol-driven)	NR

Included studies in the systematic review with exploratory synthesis of intraoperative pyloric drainage for prevention of delayed gastric conduit emptying (DGCE) after Ivor Lewis esophagectomy. *N* = 4 studies, 739 patients

*AC* adenocarcinoma, *DGCE* delayed gastric conduit emptying, *Jadad* Jadad score for randomized trials (out of 5), *NGT* nasogastric tube, *NOS* Newcastle–Ottawa Scale (out of 9), *NR* not reported, *POD* postoperative day, *RCT* randomized controlled trial, *RoB 2* Cochrane Risk of Bias 2 tool for randomized trials, *ROBINS-I* Risk Of Bias In Non-randomized Studies of Interventions, *SCC* squamous cell carcinoma, *TPN* total parenteral nutrition. The “Timing of DGCE assessment” column was added during the revision (in response to Reviewer 2) to separate the timing of the DGCE diagnosis from the overall study follow-up duration

Extracted variables included patient demographics (age, sex), tumor histology, neoadjuvant therapy, surgical approach, DGCE definition and assessment method, and follow-up duration. Non-comparative studies, animal studies, reviews, conference abstracts, editorials, non-English publications, studies with mixed surgical approaches without isolated ILE data, and studies evaluating postoperative pyloric interventions were excluded. Missing data were not imputed, and the study authors were not contacted. Funding sources and conflicts of interest were not analyzed.

The intervention of interest was defined a priori as any intraoperative prophylactic pyloric drainage technique performed during the index esophagectomy, including pyloroplasty, pyloromyotomy, intrapyloric botulinum toxin injection, finger-fracture pyloroplasty, and intraoperative balloon dilation. Preoperative endoscopic interventions (e.g., neoadjuvant botulinum toxin injection or endoscopic balloon dilation) and postoperative interventions were not eligible, in line with the predefined surgical scope of this review.

Eligible studies were required to provide a no-intervention comparator arm and to report arm-level events and denominators sufficient for synthesis. For multi-arm studies, each intervention arm was contrasted with the shared no-intervention arm using proportional comparator splitting (events and patients in the shared no-intervention arm divided by the number of intervention arms in that study; Cochrane Handbook §23.3.4); in the random-effects pooled DGCE proportion, the shared comparator arm was entered once to avoid double-counting [22–24].

Sources of clinical and methodological heterogeneity considered a priori included differences in patient population (proportion receiving neoadjuvant therapy, tumor histology), surgical approach (open, hybrid, totally MIE or RAMIE), DGCE definition (clinical, radiological, combined/algorithmic), and timing of DGCE assessment (early inpatient versus post-discharge follow-up); where data permitted, these were explored in subgroup and sensitivity analyses.

### Publication bias and risk of bias

Formal assessment of small-study effects (funnel plots, Egger’s regression) was not performed because the number of studies per pairwise comparison (*k* ≤ 3) is well below the threshold of *k* ≥ 10 generally recommended for informative interpretation of these methods [25]. The potential for publication bias is therefore acknowledged as a limitation rather than tested inferentially.

**Fig. 3:**
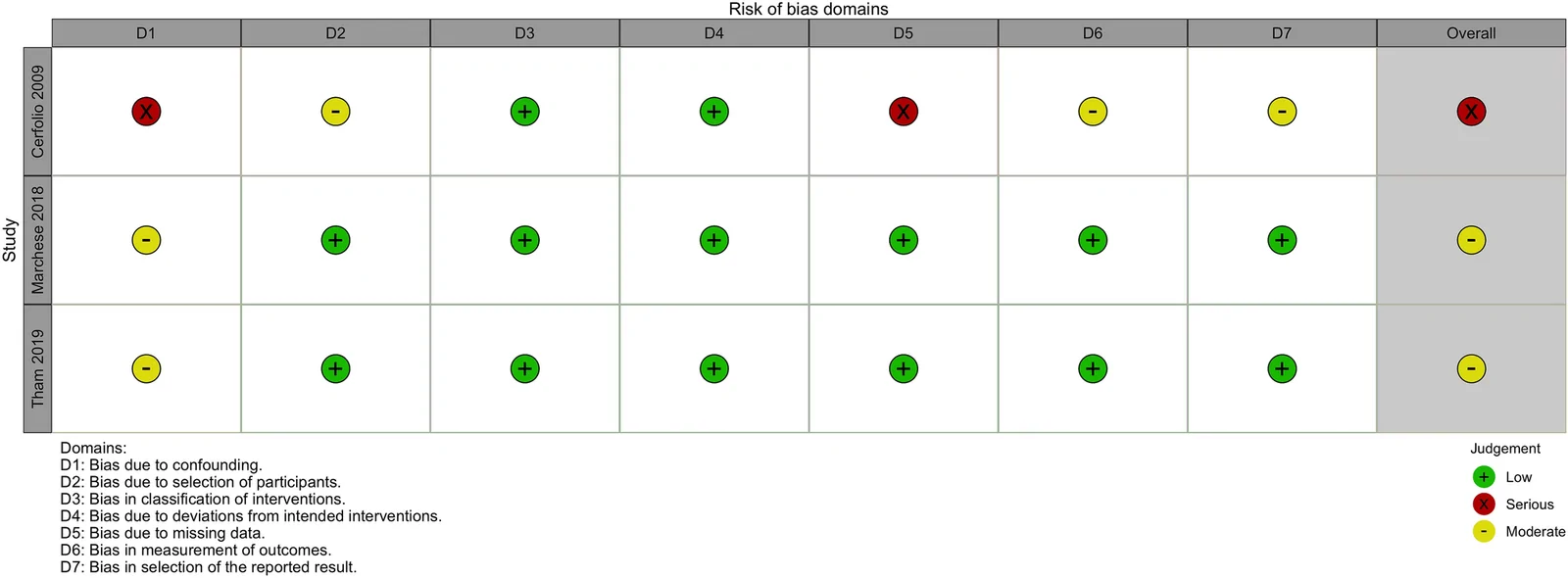
Risk-of-bias assessment of the non-randomised studies of interventions (Cerfolio 2009, Marchese 2018, Tham 2019) using the ROBINS-I tool, displayed as a traffic-light plot. Each domain is rated as low, moderate, serious, or critical risk of bias, with the overall judgement in the final column. *ROBINS-I*, Risk Of Bias In Non-randomised Studies of Interventions

**Fig. 4:**
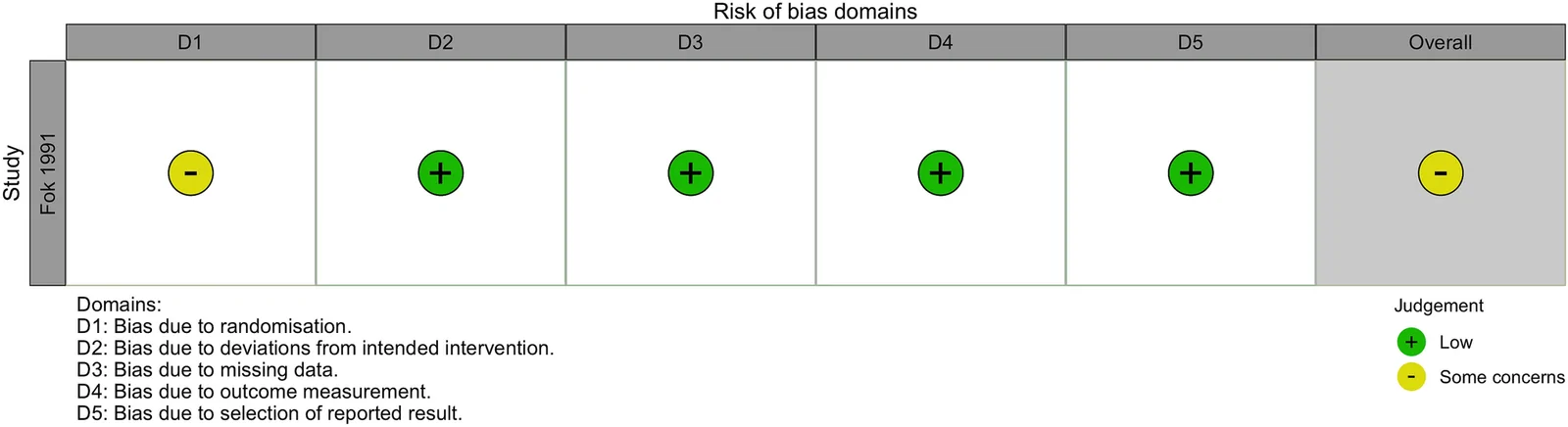
Risk-of-bias assessment of the single randomised controlled trial (Fok 1991) using the RoB 2 tool, displayed as a traffic-light plot. Domains are rated as low risk, some concerns, or high risk of bias, with the overall judgement in the final column. *RoB 2*, revised Cochrane risk-of-bias tool for randomised trials

Risk of bias was assessed independently by two reviewers (P.B., D.C.) using the ROBINS-I tool for non-randomized studies of interventions and the RoB 2 tool for randomized controlled trials [26] (Figs. 3–4). Domain-level judgements with full written justifications are provided in Supplementary Material 1; summary traffic-light plots are retained in the main text. Disagreements were resolved by consensus with a third reviewer (T.S.).

The certainty of evidence for each pairwise comparison and for the pooled DGCE proportion was evaluated using the GRADE framework as implemented in GRADEpro GDT, considering risk of bias, inconsistency, indirectness, imprecision, and publication bias [27]. Imprecision was rated against a prespecified decision threshold of an absolute DGCE rate difference of ≥ 10 percentage points (equivalently, a 95% confidence interval of the relative risk excluding both 0.80 and 1.25), reflecting the smallest difference judged clinically relevant in this surgical setting. Assessments were performed independently by two reviewers (P.B., D.C.); domain-level judgements with full justifications are reported in the Summary-of-Findings table (Table 2).

**Table 2:**
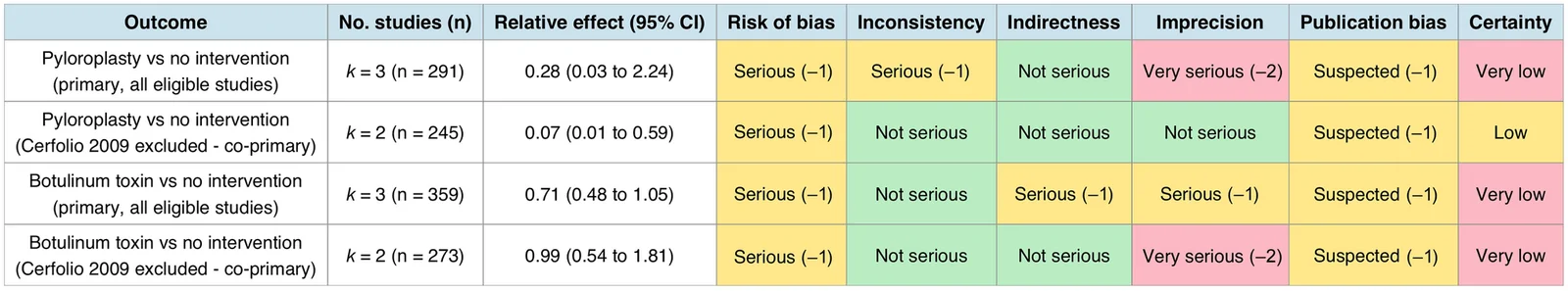
GRADE summary of findings: prophylactic intraoperative pyloric drainage versus no intervention for prevention of delayed gastric conduit emptying after Ivor Lewis esophagectomy

Imprecision was rated against a prespecified MID threshold (â‰¥ 10 percentage points absolute, equivalently a 95% CI of the relative risk excluding both 0.80 and 1.25)

Pooled DGCE incidence in the no-intervention arms (reference for absolute effects) = 32.1% (95% CI 4.4% to 82.9%; *k* = 4, *n* = 347)

## Statistical analysis

This synthesis was conducted as a structured narrative systematic review with exploratory quantitative elements, following the SWiM reporting guideline [28]. The primary mode of synthesis is a narrative summary; quantitative analyses are reported only as exploratory, hypothesis-generating estimates.

The pooled DGCE incidence in the no-intervention arms (reported as a clinical reference for the natural incidence under standard care) was estimated using a random-effects meta-analysis of single proportions, fitted as a GLMM with logit link and maximum likelihood estimation of between-study variance (τ^2^), as implemented in the R package meta [24]. For each intervention versus no intervention, exploratory random-effects pairwise RRs were estimated using inverse-variance pooling on the log-RR scale, with restricted maximum likelihood estimation of τ^2^ and Wald 95% confidence intervals; the Mantel–Haenszel estimator was also reported as the corresponding fixed-effect estimate. The Hartung–Knapp adjustment was not applied because, at *k* = 3 with substantial heterogeneity, it produced uninformatively wide intervals [25, 26]. Statistical heterogeneity was quantified using the I^2^ statistic with 95% confidence intervals and tested using Cochran’s Q. Prediction intervals are not interpreted as effect estimates, given the very small number of studies per comparison.

For each pairwise contrast in which one study used an outcome construct judged substantially different from the others, the corresponding estimate with that study omitted was reported alongside the all-study estimate as a co-primary sensitivity analysis. The two estimates are presented side by side, rather than as primary versus sensitivity, because both views are informative: the all-study estimate represents the totality of comparative evidence in the literature, whereas the Cerfolio-out estimate provides a clinically interpretable estimate based on outcome constructs that match contemporary clinical practice.

A leave-one-out sensitivity analysis was performed for every pairwise comparison to assess the influence of individual studies. Additional sensitivity analyses stratified DGCE rates by definition type (clinical, radiological, combined/algorithmic) and by timing of DGCE assessment (early inpatient versus post-discharge follow-up). Subgroup analyses were performed by study design (randomized versus observational) and intervention type. Secondary outcomes (aspiration pneumonia and other pulmonary complications, anastomotic leak, postoperative pyloric reintervention, and length of hospital stay) were extracted where reported but were not pooled due to inconsistent definitions and reporting formats; they are summarized narratively in Results.

A two-sided *p* value < 0.05 was considered statistically significant for all exploratory analyses. All analyses were performed in R version 4.5.2 (R Foundation for Statistical Computing, Vienna, Austria) using the meta package (v8.0–2) [29], metafor (v4.6–0) [30], and robvis (v0.3.0) [31].

## Results

### Study selection and patient characteristics

The systematic search identified 23,848 records, reduced to 17,070 after duplicate removal. Following title-and-abstract screening, 44 full-text articles were assessed for eligibility; 40 were excluded (Supplementary Table 1), and 4 met the prespecified criteria. The included studies comprised one prospective randomized controlled trial [32], one prospective cohort study [23], and two retrospective cohort studies [12, 22], totalling 739 patients across 11 study arms (347 no-intervention controls, 158 pyloroplasty, 163 botulinum toxin, and 71 pyloromyotomy). No eligible study evaluated intraoperative balloon dilation or finger-fracture pyloroplasty.

### Characteristics of included studies

Fok et al. (1991) enrolled 200 patients in a single-center prospective randomized controlled trial in Hong Kong, comparing Heineke–Mikulicz pyloroplasty (*n* = 100) with no intraoperative pyloric drainage (*n* = 100) during open Lewis–Tanner esophagectomy. The primary outcome was clinical gastric outlet obstruction with radiological evidence of gastric dilatation and persistent regurgitation requiring TPN [32].

Cerfolio et al. (2009) reported a single-center US retrospective cohort of 221 patients undergoing open ILE, in which four sequential intraoperative strategies were used: Heineke–Mikulicz pyloroplasty (*n* = 28), surgical pyloromyotomy (*n* = 71), no pyloric procedure (*n* = 54), and intrapyloric botulinum toxin 100 U (*n* = 68). Allocation was determined by surgeon preference and changed sequentially over time. DGCE was diagnosed radiologically on a postoperative day 4 contrast swallow study [22].

Marchese et al. (2018) reported 90 patients in a UK two-center prospective cohort allocated by center policy to no pyloric intervention (*n* = 30), intrapyloric botulinum toxin 200 U (*n* = 30), or Heineke–Mikulicz pyloroplasty (*n* = 30). DGCE was defined clinically at the first outpatient follow-up (persistent postprandial vomiting within 2 h or NGT output > 500 mL/24 h) [23].

Tham et al. (2019) analyzed 228 patients in a single-center UK before–after retrospective cohort: 163 patients operated before April 2016 received no pyloric intervention; 65 patients operated thereafter received routine intrapyloric botulinum toxin 200 U at four quadrants. DGCE was defined by an algorithm that combined NG-tube input/output and chest X-ray appearance, per the local enhanced-recovery protocol [12].

### Delayed gastric conduit emptying

As a clinical reference for the natural incidence of DGCE under standard care, an exploratory random-effects meta-analysis of single proportions restricted to the no-intervention arms (*k* = 4, *n* = 347; logit transformation, maximum likelihood) yielded a pooled DGCE incidence of 32.1% (95% confidence interval 4.4 – 82.9%) with substantial between-study heterogeneity (*τ*^2^ = 5.37, *I*^2^ = 93.7%). Crude per-study no-intervention rates ranged from 6.7% [23] to 96.3% [22].

**Fig. 5:**
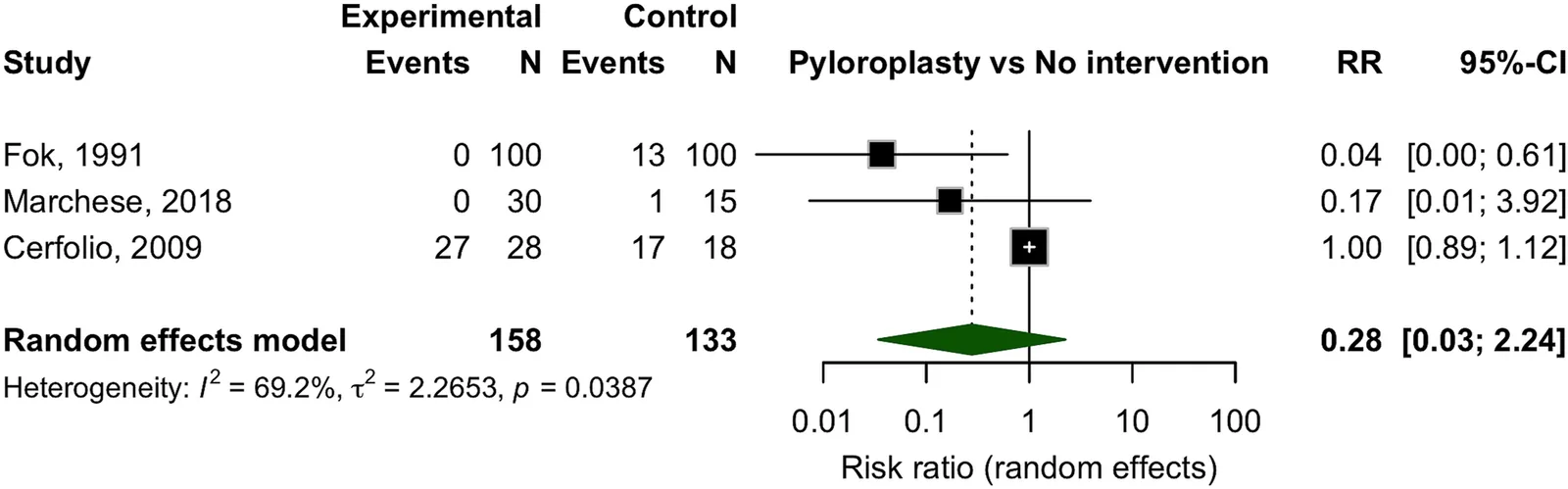
Forest plot of the exploratory random-effects meta-analysis comparing pyloroplasty with no intraoperative pyloric drainage for the incidence of delayed gastric conduit emptying (DGCE) after Ivor Lewis esophagectomy (Fok1991, Marchese 2018, Cerfolio 2009). Squares represent study-level risk ratios (RR) with 95% confidence intervals(CI); the diamond represents the pooled estimate (RR 0.28, 95% CI 0.03–2.24). Heterogeneity: I^2^ = 69.2%, τ^2^ = 2.27, p = 0.04. *DGCE*, delayed gastric conduit emptying; *RR*, risk ratio; *CI*, confidence interval

Pyloroplasty was reported in three studies [22, 23, 32]. Per-study DGCE rates diverged dramatically: 0/100 in Fok (1991) (clinical GOO requiring TPN) [32], 27/28 in Cerfolio (2009) (POD-4 radiologic) [22], and 0/30 in Marchese (2018) (clinical follow-up vomiting) [23]. The exploratory random-effects risk ratio versus no intervention was 0.28 (95% CI 0.03–2.24; *I*^2^ = 69.2%, *τ*^2^ = 2.27; *p* = 0.04 for heterogeneity); when Cerfolio (2009) was excluded as a co-primary sensitivity analysis on the basis of its incompatible POD-4 radiologic outcome construct, the pooled estimate sharpened to 0.07 (95% CI 0.01–0.59) [22], aligning with the strongly signaled benefit observed in Fok (1991) individually (Fig. 5) [32].

**Fig. 6:**
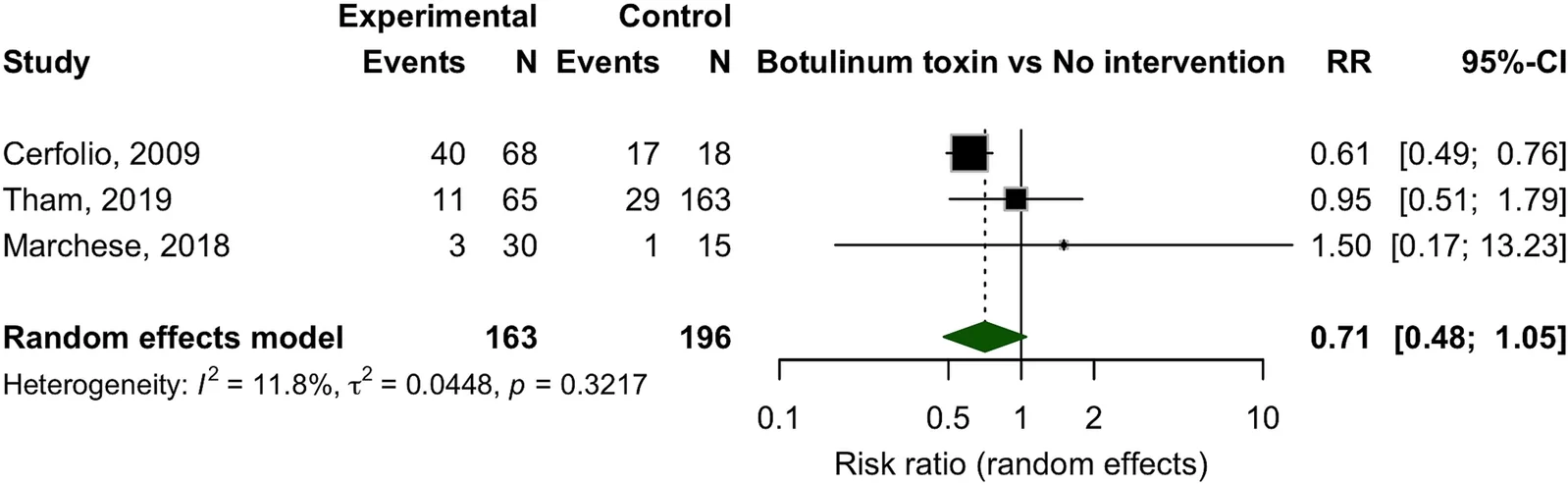
Forest plot of the exploratory random-effects meta-analysis comparing intrapyloric botulinum toxin injection with no intraoperative pyloric drainage for the incidence of delayed gastric conduit emptying (DGCE) after Ivor Lewis esophagectomy (Cerfolio 2009, Tham 2019, Marchese 2018). Squares represent study-level risk ratios (RR) with 95% confidence intervals (CI); the diamond represents the pooled estimate (RR 0.71, 95% CI 0.48–1.05). Heterogeneity: I^2^= 11.8%, τ^2^ = 0.045, p = 0.32. *DGCE*, delayed gastric conduit emptying; *RR*, risk ratio; *CI*, confidence interval

Botulinum toxin injection was reported in three studies [12, 22, 23]. Per-study DGCE rates were 40/68, 3/30, and 11/65 with botulinum toxin and 17/18, 1/15, and 29/163 with no intervention. The exploratory pooled risk ratio was 0.71 (95% CI 0.48–1.05; *I*^2^ = 11.8%, *τ*^2^ = 0.045; *p* = 0.32 for heterogeneity); when Cerfolio (2009) was excluded as a co-primary sensitivity analysis, the pooled estimate collapsed to 0.99 (95% CI 0.54–1.81), indicating that the apparent benefit signal was driven entirely by Cerfolio (2009) [22]. Marchese (2018) and Tham (2019) individually showed no benefit (Fig. 6) [12, 23].

After the eligibility criteria were applied, pyloromyotomy was reported in only a single study (Cerfolio 2009; *n* = 71), in which 66 of 71 patients (93.0%) met the radiological POD-4 DGCE definition compared with 52 of 54 patients (96.3%) in the no-intervention arm of the same study [22].

### Robustness and sensitivity analyses

Leave-one-out analyses were performed for each pairwise comparison. For the pyloroplasty contrast, removing Cerfolio (2009) increased the apparent benefit (RR 0.07, 95% CI 0.01–0.59) [22], removing Fok (1991) abolished it (RR 0.85, 95% CI 0.31–2.34) [32], removing Marchese (2018) [23] left the pooled estimate broadly unchanged (RR 0.26, 95% CI 0.01–6.28) but with widened confidence interval (Supplementary Fig. 1). For botulinum toxin, the Cerfolio-driven signal is described above (Supplementary Fig. 2). Pyloromyotomy could not be assessed (*k* = 1).

As a further exploratory check, a post hoc aggregate contrast pooling any intraoperative prophylactic drainage (pyloroplasty, botulinum toxin, or pyloromyotomy) versus no intervention yielded a relative risk of 0.83 (95% CI 0.75–0.91; I^2^ = 38.7%; *p* = 0.18 for heterogeneity). Because this contrast pools fundamentally heterogeneous interventions and is dominated by Cerfolio (2009) (133 of 167 events), it is presented only in Supplementary Fig. 3 for transparency and is not interpreted as evidence in favor of any specific technique.

Sensitivity analyses by DGCE definition for the combined pyloroplasty plus botulinum toxin contrast yielded RR 0.25 (95% CI 0.03–2.41) for clinical definitions (*k* = 3), RR 0.79 (95% CI 0.49–1.28) for radiological definitions (*k* = 2), and RR 0.95 (95% CI 0.51–1.79) for the combined/algorithmic definition (*k* = 1) (Supplementary Fig. 4). Stratification by timing of assessment showed an analogous pattern: early inpatient radiologic assessment (Cerfolio 2009) RR 0.79 (95% CI 0.49–1.28) [22]; post-discharge clinical follow-up (Marchese 2018) RR 0.68 (95% CI 0.09–5.33) [23]; and early inpatient algorithm-based assessment (Tham 2019) RR 0.95 (95% CI 0.51–1.79) (Supplementary Fig. 5) [12].

### Secondary outcomes

Pulmonary complications and aspiration pneumonia were reported with materially different definitions across studies. Fok (1991) reported 16 cardiopulmonary events versus 23 in controls (16% vs. 23%) [32]; Cerfolio (2009) reported respiratory morbidity ranging from 13% (botulinum toxin arm) to 32% (pyloroplasty arm) [22]; Marchese (2018); and Tham (2019) reported pneumonia rates broadly comparable across arms. No consistent direction of effect, favoring or disfavoring intraoperative drainage, was evident [12, 23].

Anastomotic leak was uncommon across all four studies (range 0–7% per arm) and was not consistently associated with intervention typ.

Postoperative endoscopic pyloric reintervention was needed in 11–12% of patients in both intervention and no-intervention arms of Tham (2019) and predominantly in the botulinum toxin arm of Marchese (2018) (4 inpatient and 6 outpatient endoscopic interventions, all in the botulinum toxin group; *p* = 0.032 and *p* = 0.003, respectively, versus the other arms) [12, 23].

Length of hospital stay ranged from a median of 9 days [12] (Tham (2019), botulinum toxin) to 17 days [23] (Marchese (2018), botulinum toxin); within-study differences between arms were not statistically significant in Tham (2019) [12] (median 9 vs. 10 days; *p* = 0.516) or Marchese (2018) [23].

### Certainty of evidence

Risk-of-bias judgements (Figs. 3 and 4) classified the single randomized trial (Fok 1991) as having “some concerns” under RoB 2, Cerfolio (2009) as serious under ROBINS-I (driven by confounding by indication and selective availability of the POD-4 radiologic outcome) [32], and Marchese (2018) and Tham (2019) as moderate. [12, 23] Combined with the very small number of studies per comparison, the wide confidence intervals around all exploratory effect estimates, and the marked between-study heterogeneity in DGCE definition and timing, the certainty of evidence under the GRADE framework was rated very low for both formal pairwise comparisons. The body of evidence is therefore insufficient to support firm inference about any specific intraoperative pyloric drainage technique in ILE.

## Discussion

The results of this systematic review with exploratory synthesis underscore a persistent lack of evidence regarding the role of prophylactic pyloric drainage in ILE. While pyloroplasty was numerically associated with the lowest incidence of DGCE, the overall data remain statistically inconclusive. This ambiguity likely stems from two fundamental issues: the lack of standardized diagnostic criteria and a historical oversimplification of the underlying pathophysiology.

A primary finding of this study is the profound heterogeneity in DGCE definitions across the surgical literature [12, 22, 23, 32]. Diagnostic thresholds vary from early postoperative radiologic findings [22] to late-stage clinical symptoms such as postprandial vomiting [23]. This "diagnostic anarchy" makes it difficult to determine if a drainage procedure failed or if the study simply used a more sensitive detection method. Notably, despite the standardized Delphi-derived criteria proposed by Konradsson et al. in 2020 [21], all four included studies predate this expert evidence, and no unified definition has yet been widely adopted. While this lack of standardization limits the robustness of any pooled estimate, the present synthesis remains informative as a structured narrative description of the available evidence and as a transparent demonstration of where future research is most needed.

Furthermore, the prevailing surgical hypothesis has long focused almost exclusively on pyloric spasm resulting from intraoperative vagotomy [5, 33]. However, the very high DGCE rate observed in the pyloromyotomy arm (Cerfolio (2009) was the only contributing study after eligibility was applied; 66 of 71 patients 93.0%) is dominated by that study’s POD-4 radiologic outcome definition rather than reflecting harm of pyloromyotomy itself, and a complementary explanation is selection bias in this single retrospective cohort. With these caveats, the data nevertheless support a more nuanced pathophysiological framework. As proposed in the classification by Schneider et al. (2025), DGCE should be viewed as a potential interplay between functional pyloric obstruction and mechanical conduit factors, such as redundant conduit length, axis rotation, or compression at the diaphragmatic hiatus. If the primary driver of emptying failure is mechanical rather than functional, a pyloroplasty or botulinum toxin injection may fail to improve outcomes [11].

Our findings are consistent with previous literature examining pyloric drainage during esophagectomy. Earlier systematic reviews, including Urschel et al. (2002), suggested that pyloroplasty reduced functional outlet obstruction without clear improvement in clinical endpoints [34]. A more recent meta-analysis by Loo et al. (2023) reported a reduced incidence of DGCE associated with pyloric drainage, although that analysis included heterogeneous esophagectomy techniques and outcomes [35]. By restricting inclusion to the ILE, our study reduces surgical heterogeneity and provides a more focused assessment. However, the overall evidence base remains limited: besides Fok et al. (1991), only Cheung et al. (1987) published a prospective RCT comparing pyloroplasty to no drainage during esophagectomy, both predating the MIE era [8, 32]. Recently, Luketich et al. (2025) published the first phase III randomized controlled trial of intraoperative pyloroplasty during MIE or RAMIE. The trial randomized 143 patients (134 evaluable) and was stopped early when the pyloroplasty arm reduced the composite complication rate (pneumonia and/or anastomotic leak) from 27.1 to 17.6%, a 35% relative reduction. Of the evaluable patients, 116 (87%) underwent ILE with intrathoracic anastomosis, so the trial population is predominantly an Ivor Lewis cohort. The results report only an overall between-group *p* value of 0.008 for DGCE, without arm-stratified event counts, which precludes calculation of a risk ratio. The Luketich trial nevertheless substantially shifts the contemporary evidence base in favor of routine pyloroplasty during MIE, and an Ivor Lewis-restricted analog with reported event-level data on DGCE is the most pressing outstanding research question [36].

The absence of data regarding intraoperative balloon dilation or mechanical pyloroclasy is another striking finding. Despite these being widely adopted procedures in many high-volume centers (particularly during the era of open transthoracic esophagectomy), they are virtually absent from contemporary comparative trials [37]. While the recent phase III RCT by Luketich et al. (2025) provides the first level 1 evidence supporting pyloroplasty during MIE/RAMIE, it did not evaluate these mechanical widening techniques either [36]. This “evidence gap” suggests a disconnect between traditional surgical craftsmanship and modern clinical reporting. Two complementary considerations should be noted. First, balloon dilation in contemporary practice is most often performed preoperatively as an endoscopic procedure rather than intraoperatively, which falls outside the predefined PICO of this review; the absence of intraoperative balloon-dilation studies therefore reflects a true intraoperative literature gap rather than overly restrictive eligibility [38, 39]. Second, the lack of intraoperative comparative data for mechanical pyloroclasy and finger-fracture techniques limits the present comparison to the chemical (botulinum toxin) and surgical (pyloroplasty, pyloromyotomy) interventions actually reported in eligible studies.

The limitations of the current evidence base are significant. The analysis is constrained by a small number of included studies, only one of which was a randomized controlled trial [32]. Risk of bias was rated “some concerns” under RoB 2 for the single randomized trial (Fok 1991) and moderate (Marchese 2018, Tham 2019) to serious (Cerfolio 2009) under ROBINS-I. In Cerfolio (2009), this serious rating is further compounded by calendar-time confounding: because the four sequential management strategies were introduced one after another over almost a decade, secular improvements in perioperative, anesthetic, and nutritional care and accumulating surgical experience are temporally inseparable from the intervention itself and cannot be disentangled from any apparent treatment effect. Furthermore, the sensitivity analysis showed that the trend toward benefit in the Botox group was entirely driven by a single study [22], illustrating how fragile the current "trends" in the literature truly are. The four included studies span open and hybrid esophagectomy approaches, with no eligible cohort restricted to ILE undertaken exclusively via totally MIE or RAMIE approaches, which limits generalizability to current practice. Reflecting these limitations, the GRADE certainty of evidence was rated very low across all comparisons (Table 2). Pooled estimates from the multi-arm studies (Cerfolio 2009 and Marchese 2018) rely on the proportional comparator-splitting approach used for the shared no-intervention arm, per the Cochrane Handbook; alternative splitting schemes were not explored in a separate sensitivity analysis [24]. Finally, none of the included studies reported long-term functional outcomes, in particular health-related quality of life or tolerance of adjuvant therapy, even though these endpoints are central to the clinical rationale for preventing DGCE; the present evidence base is therefore silent on the outcomes of greatest long-term importance to patients.

In conclusion, although the exploratory evidence summarized here is at best compatible with a benefit signal for pyloroplasty when the most methodologically discrepant study (Cerfolio 2009) is excluded, the very-low-certainty body of evidence does not support a routine intraoperative prophylactic pyloric drainage strategy in ILE. Future research must move beyond binary “drainage vs no drainage” comparisons and instead utilize clinically relevant classifications that account for both functional and mechanical etiologies of DGCE. Only by identifying the specific pathomechanism at play can surgeons select the appropriate intraoperative intervention, whether it be a pyloric procedure or a refinement of the conduit’s intrathoracic positioning.

## Conclusion

This systematic review with exploratory synthesis found that very-low-certainty evidence does not support routine intraoperative prophylactic pyloric drainage in ILE. Definitional heterogeneity in DGCE remains the principal methodological barrier to surgical consensus. Adequately powered, randomized trials with standardized diagnostic criteria are required, ideally informed by a pathophysiological classification that distinguishes functional from mechanical causes. At a minimum, such trials should adopt the Konradsson (2020) Delphi-based framework: an objective diagnostic threshold (> 500 mL diurnal nasogastric output on or after postoperative day 5, or a > 100% increase in conduit width with an air–fluid level on frontal chest radiography), an explicit distinction between early (≤ 14 days) and late (> 14 days) DGCE, and the accompanying standardized symptom-grading tool, so that future cohorts become quantitatively comparable and amenable to cumulative synthesis [21]. Until such evidence is available, the choice of prophylactic intervention should be individualized. The recently published phase III randomized trial during MIE argues strongly in favor of routine pyloroplasty in that setting; an Ivor Lewis-restricted analog is the most pressing outstanding research question.

## Supplementary Information

Below is the link to the electronic supplementary material.

Supplementary file1 (DOCX 16 KB)

Supplementary file2 (PNG 283 KB)

Supplementary file3 (PNG 283 KB)

Supplementary file4 (PNG 203 KB)

Supplementary file5 (PNG 523 KB)

Supplementary file6 (PNG 576 KB)

Supplementary file7 (DOCX 32 KB)

Supplementary file8 (DOCX 27 KB)

## Data Availability

All data and analytic materials (template data collection forms, data extracted from the included studies, data used for the analyses, and analytic code) are available from the corresponding author upon reasonable request. No materials are deposited in a public repository.

## Abbreviations

CI: Confidence interval
DGCE: Delayed gastric conduit emptying
GLMM: Generalized linear mixed model
GOO: Gastric outlet obstruction
GRADE: Grading of Recommendations Assessment, Development and Evaluation
ILE: Ivor Lewis esophagectomy
MeSH: Medical Subject Headings
MID: Minimally important difference
MIE: Minimally invasive esophagectomy
ML: Maximum likelihood
NGT: Nasogastric tube
PICO: Population, Intervention, Comparison, Outcome
POD: Postoperative day
PRISMA: Preferred Reporting Items for Systematic Reviews and Meta-Analyses
PROSPERO: International Prospective Register of Systematic Reviews
RAMIE: Robot-assisted MIE
RCT: Randomized controlled trial
REML: Restricted maximum likelihood
RoB 2: Risk of Bias 2 (Cochrane risk-of-bias tool for randomized trials)
ROBINS-I: Risk Of Bias In Non-randomized Studies of Interventions
RR: Risk ratio
SWiM: Synthesis Without Meta-analysis
TPN: Total parenteral nutrition
