# Supplementary material for: Prophylactic pyloric drainage for prevention of delayed gastric conduit emptying following Ivor Lewis esophagectomy: a systematic review with exploratory synthesis
Source: Surg Endosc. 2026 Jul 31;40(9):7330–41. doi: 10.1007/s00464-026-13189-3 (PMC13582249; doi:10.1007/s00464-026-13189-3)
Supplement: Supplementary file 1 [file 464_2026_13189_MOESM1_ESM.docx]

**Supplementary Appendices 1: Searchstring Pubmed**

(("Esophageal Surgery"[Mesh] OR "Esophagectomy"[Mesh] OR "Esophagus"[Mesh] OR "esophageal surgery"[Title/Abstract] OR "esophagectomy"[Title/Abstract] OR "esophagogastrectomy"[Title/Abstract] OR "esophago-gastrectomy"[Title/Abstract] OR "gastric pull-up"[Title/Abstract] OR "esophagus"[Title/Abstract] OR "ivor lewis"[Title/Abstract] OR "thoracoabdominal esophagectomy"[Title/Abstract]) AND ("Pylorus"[Mesh] OR "Botulinum Toxins"[Mesh] OR "Pyloroplasty"[Mesh] OR "Pyloromyotomy"[Mesh] OR "Erythromycin"[Mesh] OR "pylorus"[Title/Abstract] OR "pyloric drainage"[Title/Abstract] OR "pyloric drainage procedure"[Title/Abstract] OR "pyloric drainage technique"[Title/Abstract] OR "pyloric intervention"[Title/Abstract] OR "gastric drainage"[Title/Abstract] OR "pyloroplasty"[Title/Abstract] OR "pyloromyotomy"[Title/Abstract] OR "botulinum toxin"[Title/Abstract] OR "botulinum injection"[Title/Abstract] OR "botulinum A"[Title/Abstract] OR "botulinum B"[Title/Abstract] OR "chemical pyloroplasty"[Title/Abstract] OR "botox"[Title/Abstract] OR "BTX"[Title/Abstract] OR "BoNT"[Title/Abstract] OR "balloon dilatation"[Title/Abstract] OR "balloon dilation"[Title/Abstract] OR "endoscopic balloon"[Title/Abstract] OR "balloon therapy"[Title/Abstract] OR "pneumatic dilatation"[Title/Abstract] OR "finger fracture"[Title/Abstract] OR "erythromycin"[Title/Abstract] OR "prokinetic"[Title/Abstract] OR "metoclopramide"[Title/Abstract] OR "domperidone"[Title/Abstract] OR "cisapride"[Title/Abstract] OR "mosapride"[Title/Abstract]))

**Supplementary Appendices 2: Searchstring Embase**

('esophageal surgery'/exp OR 'esophagectomy'/exp OR 'esophagus'/exp OR 'esophageal surgery':ti,ab OR 'esophagectomy':ti,ab OR 'esophagogastrectomy':ti,ab OR 'esophago-gastrectomy':ti,ab OR 'gastric pull-up':ti,ab OR 'esophagus':ti,ab OR 'ivor lewis':ti,ab OR 'thoracoabdominal esophagectomy':ti,ab) AND ('pylorus'/exp OR 'botulinum toxin'/exp OR 'pyloroplasty'/exp OR 'pyloromyotomy'/exp OR 'erythromycin'/exp OR 'pylorus':ti,ab OR 'pyloric drainage':ti,ab OR 'pyloric drainage procedure':ti,ab OR 'pyloric drainage technique':ti,ab OR 'pyloric intervention':ti,ab OR 'gastric drainage':ti,ab OR 'pyloroplasty':ti,ab OR 'pyloromyotomy':ti,ab OR 'botulinum toxin':ti,ab OR 'botulinum injection':ti,ab OR 'botulinum a':ti,ab OR 'botulinum b':ti,ab OR 'chemical pyloroplasty':ti,ab OR 'botox':ti,ab OR 'btx':ti,ab OR 'bont':ti,ab OR 'balloon dilatation':ti,ab OR 'balloon dilation':ti,ab OR 'endoscopic balloon':ti,ab OR 'balloon therapy':ti,ab OR 'pneumatic dilatation':ti,ab OR 'finger fracture':ti,ab OR 'erythromycin':ti,ab OR 'prokinetic':ti,ab OR 'metoclopramide':ti,ab OR 'domperidone':ti,ab OR 'cisapride':ti,ab OR 'mosapride':ti,ab)

**Supplementary Appendices 3: Searchstring Scopus**

(TITLE-ABS-KEY("esophageal surgery" OR "esophagectomy" OR "esophagogastrectomy"

OR "esophago-gastrectomy" OR "gastric pull-up" OR "esophagus" OR "ivor lewis" OR "thoracoabdominal esophagectomy")) AND (TITLE-ABS-KEY("pylorus" OR "pyloric drainage" OR "pyloric drainage procedure" OR "pyloric drainage technique" OR "pyloric intervention" OR "gastric drainage" OR "pyloroplasty" OR "pyloromyotomy" OR "botulinum toxin" OR "botulinum injection" OR "botulinum A" OR "botulinum B" OR "chemical pyloroplasty" OR "botox" OR "BTX" OR "BoNT" OR "balloon dilatation" OR "balloon dilation" OR "endoscopic balloon" OR "balloon therapy" OR "pneumatic dilatation" OR "finger fracture" OR "erythromycin" OR "prokinetic" OR "metoclopramide" OR "domperidone" OR "cisapride" OR "mosapride"))

**Supplementary Appendices 4: Searchstring Cochrane**

("esophageal surgery" OR "esophagectomy" OR "esophagogastrectomy" OR "esophago-gastrectomy" OR "gastric pull-up" OR "esophagus" OR "ivor lewis" OR "thoracoabdominal esophagectomy") AND ("pylorus" OR "pyloric drainage" OR "pyloric drainage procedure" OR "pyloric drainage technique" OR "pyloric intervention" OR "gastric drainage" OR "pyloroplasty" OR "pyloromyotomy" OR "botulinum toxin" OR "botulinum injection" OR "botulinum A" OR "botulinum B" OR "chemical pyloroplasty" OR "botox" OR "BTX" OR "BoNT" OR "balloon dilatation" OR "balloon dilation" OR "endoscopic balloon" OR "balloon therapy" OR "pneumatic dilatation" OR "finger fracture" OR "erythromycin" OR "prokinetic" OR "metoclopramide" OR "domperidone" OR "cisapride" OR "mosapride")
