# Supplementary material for: Prophylactic pyloric drainage for prevention of delayed gastric conduit emptying following Ivor Lewis esophagectomy: a systematic review with exploratory synthesis
Source: Surg Endosc. 2026 Jul 31;40(9):7330–41. doi: 10.1007/s00464-026-13189-3 (PMC13582249; doi:10.1007/s00464-026-13189-3)
Supplement: Supplementary file 2 [file 464_2026_13189_MOESM7_ESM.docx]

Supplementary Material 1

**Risk-of-bias assessment with detailed domain-level justifications**

Risk of bias was assessed independently by two reviewers (P.B., D.C.). The ROBINS-I tool (Sterne et al., BMJ 2016) was applied to the three non-randomised studies of interventions (Cerfolio 2009, Marchese 2018, Tham 2019). The RoB 2 tool (Sterne et al., BMJ 2019) was applied to the one randomised controlled trial (Fok 1991). Disagreements were resolved by consensus with a third reviewer (T.S.). The summary traffic-light plots are retained in the main manuscript (Supplementary Figures 2 and 3); the present document provides the full domain-level judgements with written justifications for each included study, as requested by the editorial review.

# Part A — ROBINS-I assessment of non-randomised studies

## Cerfolio et al. 2009 (USA, single-centre, retrospective cohort, 4-arm sequential design)

| **Domain** | **Judgement** | **Justification** |
| --- | --- | --- |
| **D1 Confounding** | **Serious** | Allocation to the four arms was determined by surgeon preference and was sequential over the study period (pyloroplasty in the early era, pyloromyotomy and no-intervention in the middle period, intra-pyloric botulinum toxin in the most recent period). Calendar-time confounding is therefore inherent — secular changes in peri-operative management, neoadjuvant practice, ICU support, and operator experience map onto intervention status. No propensity matching, multivariable adjustment for time period, or restriction analysis is reported. This is the study’s most important limitation. |
| **D2 Selection of participants into the study** | **Moderate** | Single-centre retrospective cohort spanning approximately a decade. Eligibility criteria (Ivor Lewis esophagectomy with intrathoracic anastomosis) are explicit but the study does not report whether any patients meeting these criteria were excluded from the analysis or compare baseline characteristics of dropouts versus included patients. |
| **D3 Classification of the interventions** | **Low** | Each of the four interventions is clearly and unambiguously defined (Heineke–Mikulicz pyloroplasty; surgical pyloromyotomy; no pyloric procedure; intra-pyloric botulinum toxin 100 U in saline at four quadrants). Information used to classify intervention status is intra-operative and would have been recorded prospectively in the operative note. |
| **D4 Deviations from intended interventions** | **Low** | All interventions are performed once at the index operation. Post-operative deviations from the intended protocol (e.g., addition of endoscopic dilation) are not reported but would not, by ROBINS-I logic, attribute risk to the original allocation. |
| **D5 Missing data** | **Serious** | Missing-data risk is judged serious because the primary outcome is a POD-4 contrast swallow study; patients who did not undergo the scheduled radiological assessment (e.g., because of clinical instability, transfer, or early discharge) are not described, nor is any sensitivity analysis presented for selective availability of the outcome measurement. |
| **D6 Measurement of the outcome** | **Moderate** | DGCE is defined as radiological evidence of delayed contrast passage on a POD-4 swallow study. This is an early functional measure that does not distinguish DGCE from physiologic post-operative gastric stasis; the very high overall DGCE rates (>93 % in three of four arms) suggest that this measurement captures a different construct from the clinical DGCE measured in Marchese 2018, Tham 2019 and Fok 1991. Outcome assessor blinding is not described; assessment by a radiologist who is independent of the surgical team is plausible but is not stated. |
| **D7 Selection of the reported result** | **Moderate** | The POD-4 outcome is the only DGCE outcome reported. Longer-term outcomes (post-discharge symptoms, weight loss, late gastric outlet obstruction) that are likely to have been recorded routinely are not presented. There is no protocol pre-registration. We cannot confirm or rule out selective reporting of the most favourable comparison among multiple possible analyses. |
| **Overall risk of bias** | **Serious** | Driven by serious concerns in confounding (D1) and missing data (D5); moderate concerns in measurement of outcome (D6) and selection of reported result (D7) compound the overall judgement. The Cerfolio 2009 study is the dominant source of methodological uncertainty in the present synthesis. |

## Marchese et al. 2018 (UK, two-centre, prospective cohort, 3-arm design)

| **Domain** | **Judgement** | **Justification** |
| --- | --- | --- |
| **D1 Confounding** | **Moderate** | Two-centre prospective cohort with allocation by treating-centre policy rather than randomisation. Baseline characteristics across the three arms are broadly comparable (age, sex, neoadjuvant therapy uptake) and the cohorts were enrolled contemporaneously, which limits calendar-time confounding. No multivariable adjustment is reported, but the prospective design and contemporary enrolment reduce — without eliminating — confounding by indication. |
| **D2 Selection of participants into the study** | **Low** | Consecutive enrolment of all eligible Ivor Lewis esophagectomy patients during the study window is described; selection of participants into the cohort is therefore unlikely to have introduced bias. |
| **D3 Classification of the interventions** | **Low** | Interventions are clearly defined (Heineke–Mikulicz pyloroplasty; intra-pyloric botulinum toxin 200 U in four quadrants; no pyloric procedure) and were performed at the index operation according to the centre’s standard protocol. |
| **D4 Deviations from intended interventions** | **Low** | Surgical interventions performed at the index operation; no relevant deviations from intended treatment that would attribute risk to the assigned arm. |
| **D5 Missing data** | **Low** | Outcome was assessed at the first scheduled outpatient follow-up; follow-up rates are reported and are similar across arms. |
| **D6 Measurement of the outcome** | **Low** | DGCE was defined clinically (post-prandial vomiting within 2 h or NGT output >500 mL/24 h) at the first outpatient follow-up, approximately two to three weeks after discharge. Outcome ascertainment is uniform across arms; assessor blinding is not described, but the objectivity of the NGT-output criterion limits the impact of any unblinded assessment. |
| **D7 Selection of the reported result** | **Low** | All pre-specified outcomes appear to be reported. No protocol pre-registration is cited, but the analysis plan is consistent with what would be expected from a prospective cohort of this size. |
| **Overall risk of bias** | **Moderate** | Driven primarily by residual confounding by centre-level preference (D1); other domains are at low risk of bias. |

## Tham et al. 2019 (UK, single-centre, retrospective cohort, before–after design)

| **Domain** | **Judgement** | **Justification** |
| --- | --- | --- |
| **D1 Confounding** | **Moderate** | Single-centre retrospective cohort with a before–after design: intra-pyloric botulinum toxin (200 U at four quadrants) was introduced as routine practice from April 2016 onwards, while patients operated on before this date received no pyloric intervention. Allocation is therefore largely determined by calendar period rather than by individual patient selection, which limits patient-level confounding by indication but introduces calendar-time confounding (changes in peri-operative pathway, ICU practice, or surgical experience over the study period). Baseline comparisons between groups (age, sex, neoadjuvant therapy, tumour histology) are broadly similar and reported. |
| **D2 Selection of participants into the study** | **Low** | Consecutive enrolment of all Ivor Lewis esophagectomy patients operated within the time window; no exclusion of subgroups based on outcome or post-operative course is described. |
| **D3 Classification of the interventions** | **Low** | Interventions are clearly defined (intra-pyloric botulinum toxin endoscopic at four quadrants; no pyloric intervention), and the intra-operative records used for classification are unambiguous. |
| **D4 Deviations from intended interventions** | **Low** | Surgical interventions at the index operation, performed as planned. |
| **D5 Missing data** | **Low** | Within-admission outcome assessment with explicit algorithm-based criteria; missing-data risk is low. |
| **D6 Measurement of the outcome** | **Low** | DGCE was defined by an algorithm combining NG-tube input/output differences and chest X-ray appearance according to a written enhanced-recovery protocol (Table 1 of the original paper). Outcome assessment is therefore semi-objective and applied uniformly across arms. |
| **D7 Selection of the reported result** | **Low** | All pre-specified outcomes appear to be reported. No protocol pre-registration is cited. |
| **Overall risk of bias** | **Moderate** | Driven by calendar-time confounding (D1) inherent to the before–after design; other domains are at low risk. |

# Part B — RoB 2 assessment of the randomised controlled trial

## Fok et al. 1991 (Hong Kong, single-centre, prospective RCT)

| **Domain** | **Judgement** | **Justification** |
| --- | --- | --- |
| **D1 Bias arising from the randomisation process** | **Some concerns** | Patients were prospectively randomised between pyloroplasty and no-drainage groups. The randomisation method is not described in detail, and allocation concealment is not explicitly addressed in the published report. Baseline characteristics are reasonably balanced, but the limited reporting prevents full confidence in the randomisation process by current standards. |
| **D2 Bias due to deviations from intended interventions** | **Low** | Both groups received the index esophagectomy as intended. Pyloroplasty (Heineke–Mikulicz with continuous suture) was performed at the time of esophagectomy in the experimental arm; no pyloric procedure was performed in the control arm. No post-randomisation cross-over or unintended deviations are reported. |
| **D3 Bias due to missing outcome data** | **Low** | Outcome was assessed in the index hospital admission and at regular outpatient follow-up; missing-data risk is low. |
| **D4 Bias in measurement of the outcome** | **Low** | DGCE was defined as clinical gastric outlet obstruction with radiological evidence of gastric dilatation and persistent regurgitation requiring TPN. Outcome ascertainment was uniform across arms; assessor blinding is not explicitly described, but the objective outcome criteria (need for TPN, radiological dilatation) limit the scope for differential mis-classification. |
| **D5 Bias in selection of the reported result** | **Low** | Pre-specified primary outcomes are reported; no evidence of selective outcome reporting. No protocol pre-registration is cited, in line with publication conventions of the era. |
| **Overall risk of bias** | **Some concerns** | Driven by limited reporting of the randomisation process (D1); other domains are at low risk of bias. |
