# Supplementary material for: Prophylactic pyloric drainage for prevention of delayed gastric conduit emptying following Ivor Lewis esophagectomy: a systematic review with exploratory synthesis
Source: Surg Endosc. 2026 Jul 31;40(9):7330–41. doi: 10.1007/s00464-026-13189-3 (PMC13582249; doi:10.1007/s00464-026-13189-3)
Supplement: Supplementary file 3 [file 464_2026_13189_MOESM8_ESM.docx]

**Supplementary Table 1. Excluded Full-Text Articles**

| **#** | **Study** | **Year** | **Title** | **Reason for exclusion** |
| --- | --- | --- | --- | --- |
| 1 | Bagheri et al. | 2013 | Botulinum toxin for prevention of delayed gastric emptying after esophagectomy | No isolated ILE data |
| 2 | Bolger et al. | 2020 | Gastric emptying and gastrointestinal transit after esophagectomy with pyloroplasty | No isolated ILE data |
| 3 | Bolger et al. | 2023 | Omission of intraoperative pyloric procedures in minimally invasive esophagectomy | No isolated ILE data |
| 4 | Burt et al. | 1996 | Erythromycin stimulates gastric emptying after esophagectomy with gastric replacement | No isolated ILE data |
| 5 | Chung et al. | 2014 | Endoscopic pyloromyotomy for post-esophagectomy gastric outlet obstruction | Postoperative intervention |
| 6 | De Pasqual et al. | 2021 | Effect of pyloroplasty on gastric conduit emptying and quality of life after Ivor Lewis esophagectomy | Non-comparative study |
| 7 | Decker et al. | 2020 | Gastric emptying after esophagectomy: comparing single therapy versus dual therapy pyloric drainage | No isolated ILE data |
| 8 | Eldaif et al. | 2013 | Intrapyloric botulinum injection increases postoperative esophagectomy complications | No isolated ILE data |
| 9 | Ericson et al. | 2013 | Large-diameter pneumatic balloon dilatation of the pylorus after esophagectomy | Postoperative intervention |
| 10 | Hadzijusufovic et al. | 2019 | Preoperative endoscopic pyloric balloon dilatation decreases rate of delayed gastric emptying | No isolated ILE data |
| 11 | Hill et al. | 1993 | Gastric emptying following oesophagectomy | No isolated ILE data |
| 12 | Johansson et al. | 2000 | Anastomotic diameters and strictures following esophagectomy and total gastrectomy | No isolated ILE data |
| 13 | Kent et al. | 2007 | Pilot study of botulinum toxin injection for treatment of delayed gastric emptying following esophagectomy | No isolated ILE data |
| 14 | Letter to the Editor | 2018 | Pyloric drainage and delayed gastric emptying after esophagectomy (Chirurgia) | No isolated ILE data |
| 15 | Li et al. | 2014 | Delayed gastric emptying after esophagectomy for malignancy | No isolated ILE data |
| 16 | Ludwig et al. | 2001 | A prospective evaluation of dietary status and symptoms after near-total esophagectomy without gastric emptying procedure | No isolated ILE data |
| 17 | Maus et al. | 2016 | Gastric outlet obstruction after esophagectomy: retrospective analysis of the effectiveness and safety of postoperative endoscopic pyloric dilatation | Postoperative intervention |
| 18 | Mistry et al. | 2012 | Effect of short-term versus prolonged nasogastric decompression on major postesophagectomy complications | No isolated ILE data |
| 19 | Oezcelik et al. | 2011 | Circular stapled pyloroplasty during esophagectomy with gastric pull-up | Non-comparative study |
| 20 | Ramirez et al. | 2022 | Endoscopic pyloromyotomy in minimally invasive esophagectomy: a novel approach | No isolated ILE data |
| 21 | Stewart et al. | 2017 | Is chemical pyloroplasty necessary for minimally invasive esophagectomy? | No isolated ILE data |
| 22 | Tanaka et al. | 1996 | Emptying of the intrathoracic stomach using three different pylorus procedures | No isolated ILE data |
| 23 | Tcherniak et al. | 2006 | Treatment of gastroparesis after total esophagectomy using botulinum toxin | Postoperative intervention |
| 24 | Zhang et al. | 2017 | Risk factors for delayed gastric emptying in patients undergoing esophagectomy without pyloric drainage | Non-comparative study |
| 25 | Luketich et al. | 2025 | A Phase III Randomized Controlled Trial of Pyloroplasty versus No Pyloroplasty in Patients Undergoing Minimally Invasive Esophagectomy or Robot-Assisted Minimally Invasive Esophagectomy | No isolated ILE data |
| 26 | Saeed et al. | 2024 | Intra-operative pyloric BOTOX injection versus pyloric surgery for prevention of delayed gastric emptying after esophagectomy (Surgical Endoscopy) | Non-comparative study |
| 27–40 | Various (14 conference abstracts) | 2012–2024 | Conference abstracts and proceedings from AGA, BJS, EAES, ISDE, SAGES, SSO, STS, and others | Conference abstract |
